# MDCT-Based Finite Element Analysis for the Prediction of Functional Spine Unit Strength—An In Vitro Study

**DOI:** 10.3390/ma14195791

**Published:** 2021-10-03

**Authors:** Nithin Manohar Rayudu, Thomas Baum, Jan S. Kirschke, Karupppasamy Subburaj

**Affiliations:** 1 Engineering Product Development (EPD) Pillar, Singapore University of Technology and Design (SUTD), Singapore 487372, Singapore; rayudu_nithin@mymail.sutd.edu.sg; 2 Department of Diagnostic and Interventional Neuroradiology, School of Medicine, Klinikum rechts der Isar, Technical University of Munich, Ismaninger St. 22, 81675 Munich, Germany; thomas.baum@tum.de (T.B.); jan.kirschke@tum.de (J.S.K.); 3 Changi General Hospital, 2 Simei St. 3, Singapore 529889, Singapore; 4 Sobey School of Business, Saint Mary’s University, 903 Robie St., Halifax, NS B3H 3C2, Canada

**Keywords:** finite element analysis, multidetector computed tomography, functional spine unit, bone strength

## Abstract

(1) Objective: This study aimed to analyze the effect of ligaments on the strength of functional spine unit (FSU) assessed by finite element (FE) analysis of anatomical models developed from multi-detector computed tomography (MDCT) data. (2) Methods: MDCT scans for cadaveric specimens were acquired from 16 donors (7 males, mean age of 84.29 ± 6.06 years and 9 females, mean age of 81.00 ± 11.52 years). Two sets of FSU models (three vertebrae + two disks), one with and another without (w/o) ligaments, were generated. The vertebrae were segmented semi-automatically, intervertebral disks (IVD) were generated manually, and ligaments were modeled based on the anatomical location. FE-predicted failure loads of FSU models (with and w/o ligaments) were compared with the experimental failure loads obtained from the uniaxial biomechanical test of specimens. (3) Results: The mean and standard deviation of the experimental failure load of FSU specimens was 3513 ± 1029 N, whereas of FE-based failure loads were 2942 ± 943 N and 2537 ± 929 N for FSU models with ligaments and without ligament attachments, respectively. A good correlation (ρ = 0.79, and ρ = 0.75) was observed between the experimental and FE-based failure loads for the FSU model with and with ligaments, respectively. (4) Conclusions: The FE-based FSU model can be used to determine bone strength, and the ligaments seem to have an effect on the model accuracy for the failure load calculation; further studies are needed to understand the contribution of ligaments.

## 1. Introduction

The spine is one of the most important structures in the human axial skeleton, as it provides both mechanical support and motion flexibility to the upper part of the body [1]. The functionality of the spine is affected by multiple medical conditions such as osteoporosis (OP) with vertebral compression fractures (VSFs) [2], disk degeneration [3], or trauma [4]. Osteoporosis is a prevalent bone-related disorder that can result in excessive bone loss and microstructure deterioration. Studies have shown that the second most osteoporotic fragility fractures occur in the spine after the hip region [5]. Untreated osteoporosis can lead to vertebral fractures and, in turn, impaired quality of life and permanent disability [6]. Therefore, it is crucial to diagnose osteoporosis at an earlier stage to initiate therapy.

Clinically, bone mineral density (BMD)-based quantitative measures are being used to assess bone health and strength. Dual-energy X-ray absorptiometry (DXA)-based aerial bone mineral density (aBMD) measures, in the form of T- and Z-scores, are widely used and recognized as the standard for diagnosing osteoporosis in clinical settings [7,8]. However, studies have shown that the fracture predictive ability of the measure is under 50 percent [9]. In some cases, the patients with a healthy T-score range have suffered fragility fractures and vice versa [10]. Researchers at the University of Sheffield had developed the fracture risk assessment (FRAX) tool to estimate the probability of occurrence of fracture in the next 10 years. FRAX considers BMD at the femoral neck and twelve other clinical risk factors. Even though FRAX is an easily accessible web-based tool, it has multiple limitations reducing its efficiency [11]. Volumetric BMD (vBMD) measures derived from quantitative computed tomography (qCT) have been demonstrated for bone health assessment [12]. A recent study has shown that baseline and follow-up vBMD values at the lumbar region were able to differentiate healthy subjects and subjects with pre-existing or osteoporotic vertebral fractures [13]. Even though BMD-based measures are widely used for OP diagnosis in clinics, their ability is limited for fracture prediction. The BMD measures provide quantitative information about bone structure only. To accurately assess overall bone health and quality, it is essential to consider other factors, including spatial distribution of bone mass, morphology, structural integrity, and loading characteristics. In in vivo scenarios, the health and quality of the bone alone do not define bone strength; interactions with other bones, muscles, ligaments, and tendons contribute significantly to its functional and structural characteristics. 

The finite element (FE) method is a numerical technique that solves the partial differential equation by discretizing the model into small elements. The use of the FE method in biomechanical applications has been increased significantly over the last decade [14,15,16,17]. In the FE method, tissues of interest are segmented from radiological images, and patient-specific 3D models are generated from those segmented regions, then meshes are generated from those 3D models with optimal density, and material properties are applied to the elements in the FE mesh based on image intensity values. Finally, the loading and boundary conditions are applied to the meshed model before being solved to predict biomechanical characteristics. Bone is a complex structure, and it exhibits different material behaviors such as (1) nonlinearity [15]— bone undergoes both elastic and plastic deformation before failure; (2) inhomogeneity [15]—the bone structure and the amount of bone mass present at each location is different from other; (3) anisotropy [18]—based on the anatomical location and biomechanical loading, the human bone has evolved to handle higher loads in specific directions and considerably less in the other directions. FE methods have been demonstrated to capture and analyze these material behaviors.

FE method has been used for analyzing the vertebral bodies [17,18,19,20]. Studies have shown that FE-predicted failure loads have a higher correlation with the experimental loads and perform better than BMD-based measures in identifying patients at the risk of having bone fracture [17,21]. However, the spine is a complex biomechanical structure, with multiple interacting and connecting tissues. Thus, analyzing the single vertebra to understand the characteristics of the spine biomechanics is not sufficient. Functional Spinal units (FSUs) have been developed as a building block of the spine to understand its biomechanics and compute bone strength with higher accuracy. Anitha et al. have shown that models with intervertebral discs can predict the vertebral failure loads accurately than single vertebra models [22]. Groenen et al. applied the isotropic nonlinear material properties and modeled the FSU with three vertebrae and discs and demonstrated that the FE-predicted stiffness correlates well with experiments [23]. Xiao et al. modeled the L4-L5 lumbar FSU using the isotropic nonlinear patient-specific material properties to study its biomechanics and validated the range of motion (ROM) with experimental results [24]. Jhang et al. studied the effect of pedicle-based dynamic stabilization systems on the L4-L5 FSU FE model [25]. In comparison, significant attention has been paid to demonstrating the importance of the FSU model and its applicability in studying the kinematics of the spine, much less focus given on calculating the failure load and assess the fracture risk. Considering the FSU models consider interactions between different spine elements, the derived failure load values are of high significance to clinicians regarding spine bone strength and quality, which, in turn, helps them provide better diagnosis and treatment.

Thus, the scope of the current work was to study the feasibility of using FSU models to calculate the failure loads using finite element methodology. To achieve this goal, we set out to investigate the following objectives:

(1) Compare the failure loads of FE-based FSU models (with and without ligaments) with the experimental loads;
(2) Compare the failure loads between those with and without ligament FSU models.

## 2. Materials and Methods

**Figure 1:**
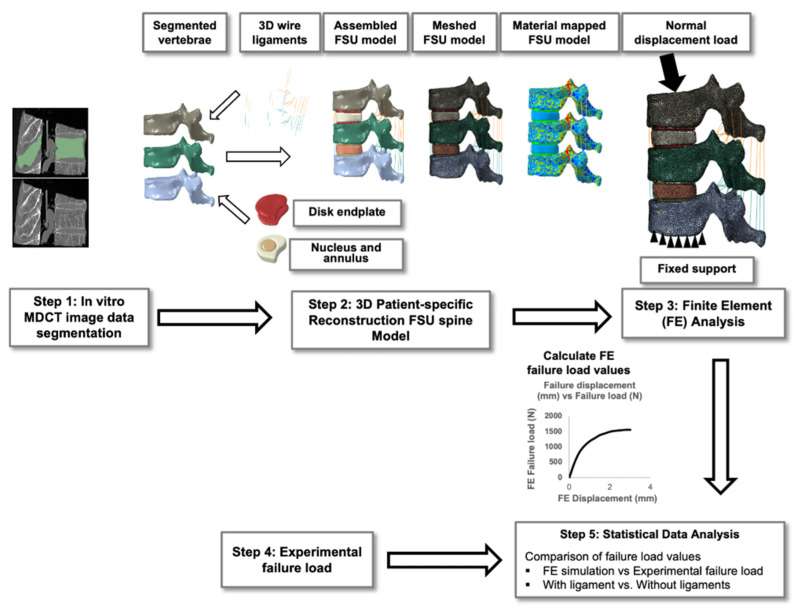
The five-step methodology followed in the current finite element study for the calculation of the failure load for the functional spine unit.

The study followed a five-step methodology. In the first step, we performed the invitro MDCT data segmentation. We reconstructed 3D anatomical models from the segmented data and developed the FSU models in the second step. In the third step, we mapped material properties, applied loading and boundary conditions, simulated the model, and calculated the failure load. We determined the experimental failure loads of those specimens from the uniaxial compression test in the fourth step. In the final step, we performed the statistical data analysis. The overall methodology is shown in detail in Figure 1.

### 2.1. Subject Data

A total of 16 specimens were taken from donors (7 males, mean age 84.29 ± 6.06 years and 9 females, mean age of 81.00 ± 11.52 years) had been included in the study. For 13 patients, the middle vertebrae of the FSU were present in the thoracic region (T10 vertebrae for 6 patients; T9 vertebrae for two patients; T12 vertebrae for two patients; T6 for one patient; T8 for one patient; and T11 vertebrae for one patient), and for 3 FSUs, the middle vertebrae were in the lumbar region (L3 vertebrae for two patients; and L1 vertebrae for one patient). All donors had no medical conditions such as bone metastases, as well as hematological or metabolic disorders. Before death, all the donors had donated their bodies for educational and research purposes to the local institute of anatomy. The current study, including the experimental protocol, was approved by the institutional committee for human research at the Technical University of Munich (27/29 S-SR) in accordance with the 1964 Helsinki declaration and its later amendments. 

All the FSUs with middle vertebrae, adjacent IVDs, and half of the adjacent vertebrae were taken from formalin-fixed human donors. The FSUs were extracted using the bandsaw. The muscle and fat tissue surrounding the FSUs were removed entirely, and the ligaments and posterior elements were kept intact. For avoiding any decomposition, all the FSU models were kept in formalin solution for the entire duration of the study. The procedure was performed as reported previously [22,26].

### 2.2. MDCT Image Acquisition

Invitro multidetector computed tomography (MDCT) images of these specimens were acquired with a 256-row CT scanner (iCT, Philips Medical Care, Best, The Netherlands). Scanner settings are as follows: tube load—585 mAs, tube voltage—120kVp, field view—150 mm, image pixel matrix size—1024 × 1024, and spatial resolution—250 × 250 × 600 µm^3^. For calibration purposes, during data acquisition, a phantom (Mindways Osteoporosis Phantom, San Francisco, CA, USA) was placed beneath the MDCT scanner. Additionally, a high-resolution bone kernel (YE) was used for the reconstruction of all the transverse sections.

### 2.3. Image Segmentation and 3D Reconstruction

**Figure 2:**
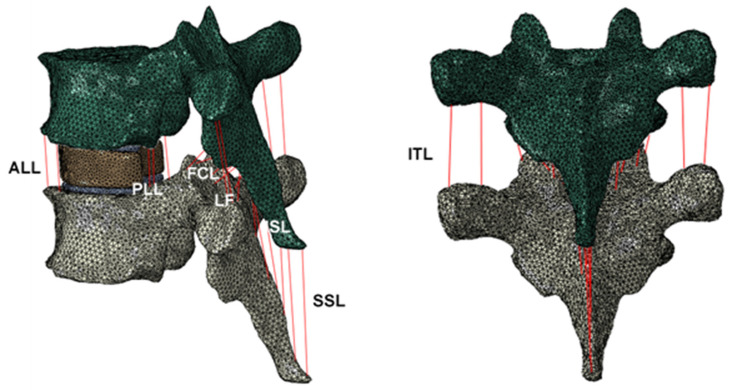
Three-dimensional truss ligaments modeled in the current finite element analysis. Anterior longitudinal ligament (ALL), posterior longitudinal ligament (PLL), ligamentum flavum (LF), intraspinous ligament (ISL), supraspinous ligament (SSL), intertransverse ligament (ITL), and facet capsular ligament (FCL).

The in vitro MDCT scan data were then imported to open-source medical image analysis software 3D slicer (https://www.slicer.org/) for 3D reconstruction of the vertebrae. The middle vertebrae contour was accurately segmented using the semiautomatic process, and a 3D model was generated. The middle vertebra was then imported to commercial finite element analysis software Abaqus v6.10 (Hibbitt, Karlsson, and Sorensen, Inc., Pawtucket, RI, USA), and the model was meshed using linear tetrahedral elements (C3D4). Then, the meshed model was imported to material mapping software Bonemat v3.2 (http://www.bonemat.org/). The image-intensity-based material properties were mapped to the vertebral model based on the empirical material mapping relations (Table 1). The material-mapped model was then imported to Abaqus. For modeling and anchoring of the ligaments, the half top and bottom vertebrae were not sufficient, so using the instances and translate option, the top and bottom vertebrae were created based on the middle vertebrae. As the mechanical properties of the adjacent vertebrae are not significantly different, we assumed it would not affect the results to a greater extent [21,27]. The translation distance between the vertebrae was determined by the distance between the vertebrae from MDCT images in the 3D slicer. Then, the disk was manually created and later divided into nucleus and annulus regions. The nucleus area was maintained to 30% of the overall disk surface area [22,28]. Then, using extrude-cut and -subtract options, endplates were created, and their thickness was kept at 1 mm. All the 3D models were then assembled and based on the anatomical location, wire elements for ligaments were modeled. We considered seven important ligaments—namely, anterior longitudinal ligament (ALL), posterior longitudinal ligament (PLL), ligamentum flavum (LF), interspinous ligament (ISL), supraspinous ligament (SSL), intertransverse ligament (ITL), and facet capsular ligament (FCL). Figure 2 shows the ligament locations. To approximately replicate the real-life tissue structure, we modeled the ligaments with multiple elements, i.e., ALL, PLL, and LF were modeled with 3; ISL and ITL were modeled with 4; SSL was modeled with 2; FCL was modeled with 6. Additionally, to avoid stress concentration, the end of each ligament was connected to multiple nodes of the vertebrae through tie constraint. Table 2 shows the ligament number, properties, and correctional area used in the current study. 

**Figure 3:**
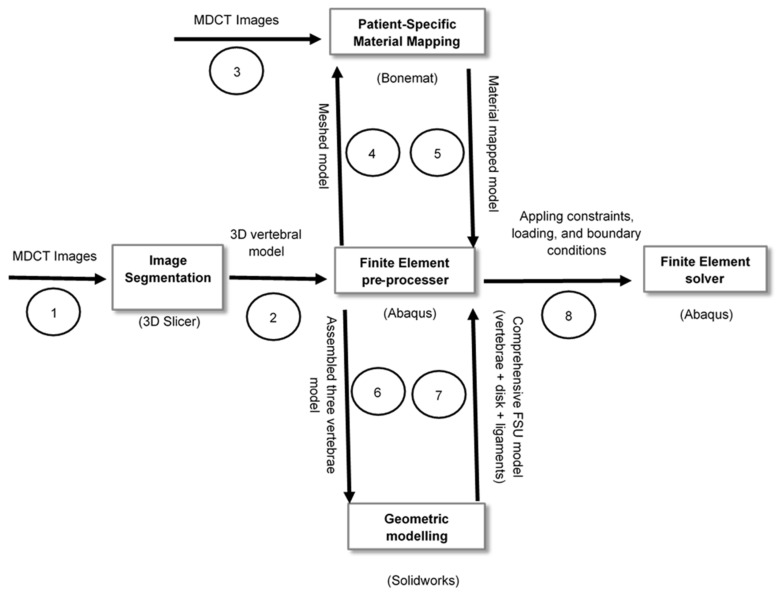
The flow of the segmented model between the computational tools from segmentation to model solving in the current analysis.

Figure 3 shows the overall flow of the model from MDCT images to solving the FE model. In the first step, the MDCT images were imported to an image segmentation tool, and a 3D vertebral model was generated. Next, this 3D model was imported to FE preprocessor software, and meshing was performed. The meshed model was sent to a material mapping tool, and the patient-specific material properties were applied. Next, the model was sent back to the FE preprocessor tool and the geometric modeling software to generate intervertebral discs, ligaments, and for the model assembly. Then, the constraints, loading, and boundary conditions were given in the FE preprocessor tool. Subsequently, the model was sent to FE Solver for analysis.

For maintaining the accuracy of the computational model, we carried out a mesh sensitivity study. We considered the mesh maximum edge sizes from 0.5 mm to 2.0 mm, with an increment of 0.25 mm, and found that 1 mm edge was producing mesh-independent results; the same had been used for the meshing of vertebrae and the disk components.

### 2.4. Simulation and Modeling

The fully assembled model was then imported to Abaqus software for further analysis. The disk and annulus were then meshed with linear tetrahedral elements, and the ligaments meshed with circular 3D truss elements (T3D2) [37]. For replicating the actual spine behavior, a tie constraint was applied between the endplate and vertebrae, between endplate and nucleus and annulus, and also between nucleus and annulus [24]. Finally, the ligaments were also connected to the vertebrae using the tie constraint. No penetration contact conditions were applied to avoid penetration in the posterior elements. For calculating the failure load, compression loading condition was simulated by fixing the inferior surface of the bottom vertebrae, and normal displacement load was applied on the superior surface of the top vertebrae. The peak of the load–displacement curve was considered as the failure load [21,22,38,39].

### 2.5. Experimental Setup

Resin (Rencast Isocyanat and Polyol, Huntsman Group, Bad Sackingen, Germany) was used to embed the top and bottom vertebrae, and it covered up to 2 mm above and 2 mm below the half vertebral bodies. For simulating the perfect axial loading conditions, parallel alignment was performed for the upper, middle, and lower vertebrae. The outer resin was also chocked to avoid any movement during the uniaxial testing. All the FSUs were fixed to the mechanical testing system (Wolpert Werkstoff- prufmaschinen AG, Schaffhausen, Switzerland). After preconditioning was carried out similar to the previous studies [40], a monotonic and uniaxial compression test was performed, and the load–displacement curve was recorded, and the FSU experimental failure load was calculated. More details about the experimentation were discussed in detail in our previous study [22]. 

### 2.6. Statistical Data Analysis

All the statistical tests were carried out using IBM SPSS Statistics for Windows (v25.0; IBM Corp., Armonk, NY, USA) and Microsoft Excel (v16.27 (2019); Microsoft Corporation, Redmond, WA, USA). Using Kolmogorov–Smirnov test, we checked the normality of the data. Additionally, we created box plots with standard deviation for comparing the FE-based failure loads of FSU models with and without ligament versus experimental failure loads. Additionally, for observing variation between them, we generated the correlation plots, and to observe the data spread, we used Bland–Altman (BA) plots [41]. Finally, we also studied the variations between with and without ligament models using the correlation and BA plots. The Wilcoxon signed-rank test was used to compare the datasets, and *p* value of < 0.01 was considered as significant for all the statistical tests. 

## 3. Results

### 3.1. Comparison of FE-Predicted FSU Failure Load Values with Experimental Results

**Figure 4:**
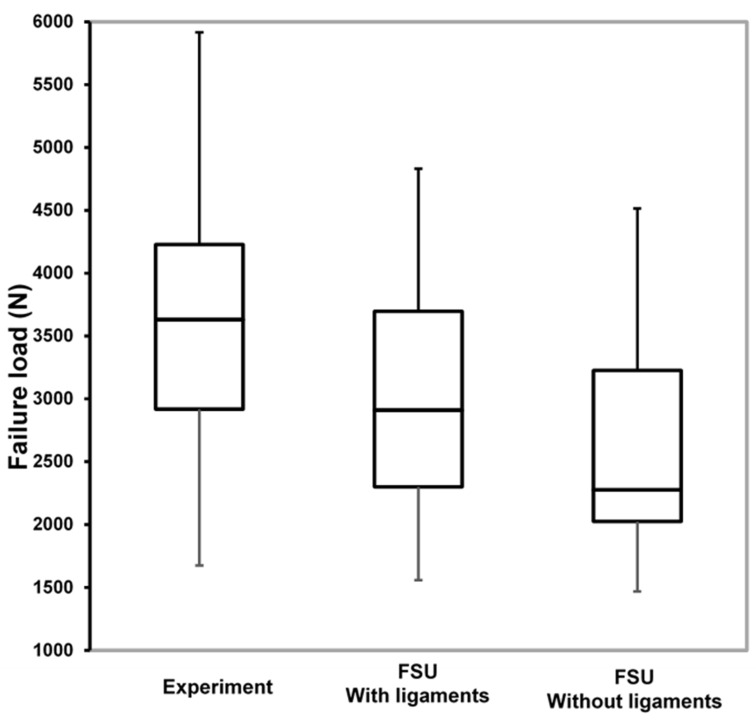
The failure load comparison box plots between the experimental, FSU with ligaments, and FSU without ligaments models.

**Figure 5:**
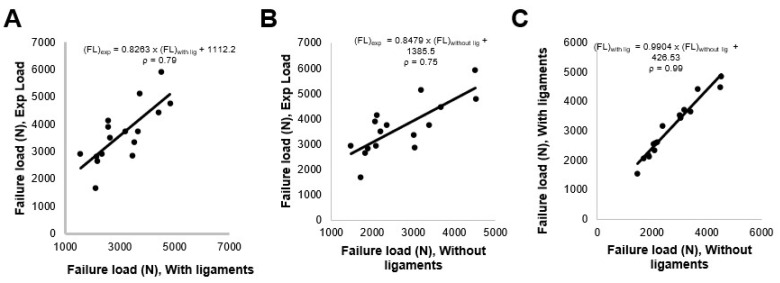
The Correlation plots: (**A**) between the experimental loads and FE failure loads for FSU with ligaments models; (**B**) between the experimental loads and FE failure loads for FSU without ligaments models; (**C**) between the FE failure loads for FSU with ligaments models and FSU without ligaments models.

**Figure 6:**
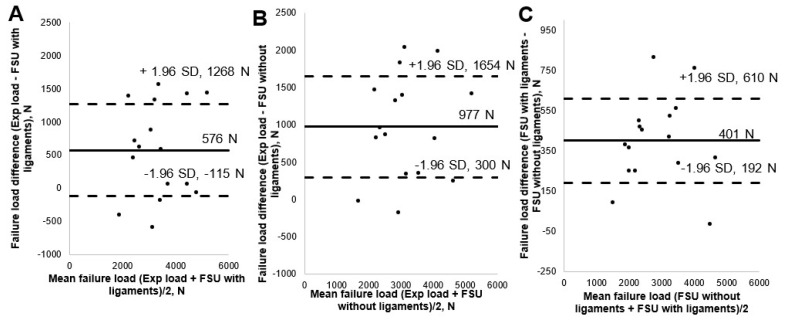
The failure load data spread plots: (**A**) between the experimental and FSU with ligaments; (**B**) between the experimental and FSU without ligaments; (**C**) between the FSU with and without ligament models. The limits of agreement are shown with dashed horizontal lines above and below the horizontal solid line indicating the mean value (±1.96 SD).

**Figure 7:**
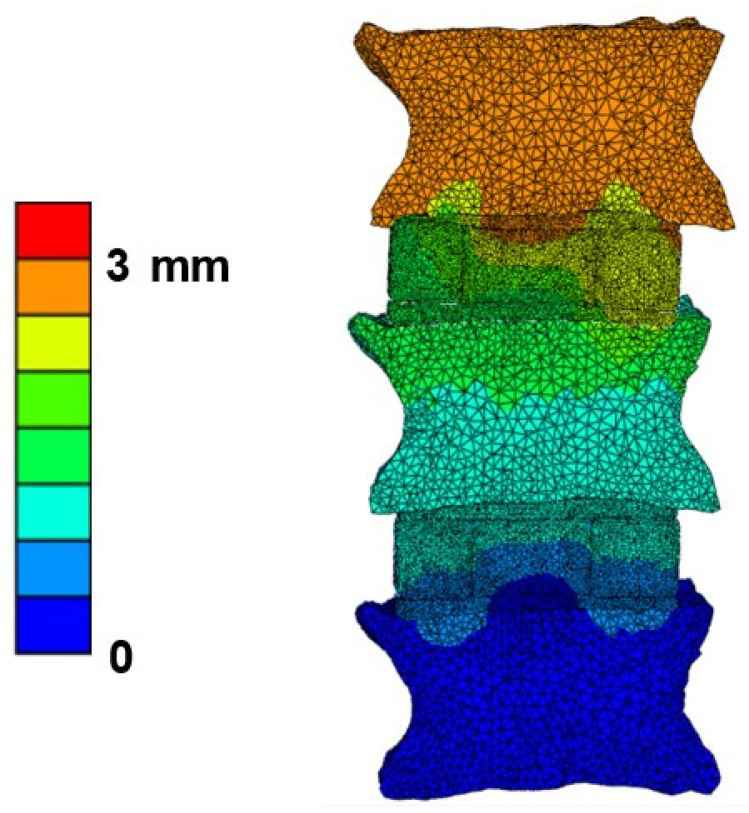
Cross-sectional view of vertical axial displacement distribution for the FSU model at failure.

**Figure 8:**
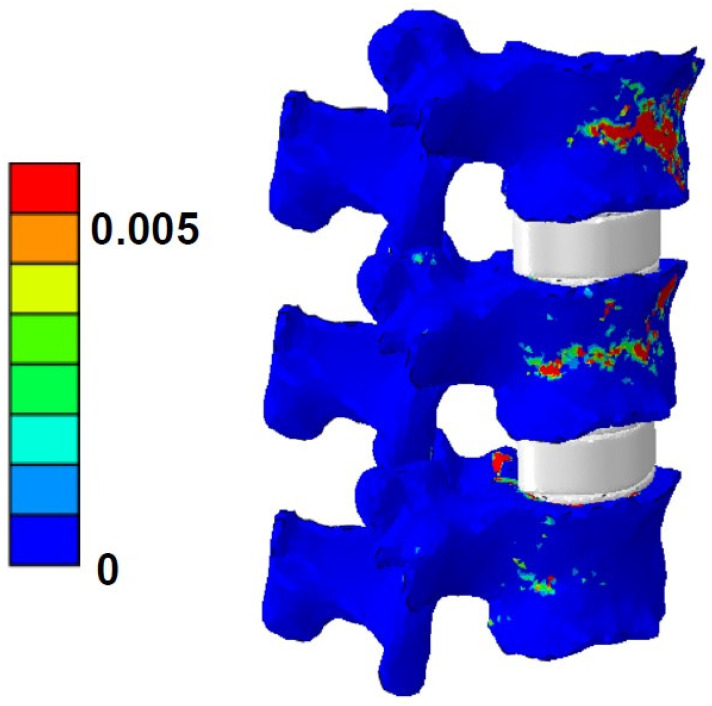
Plastic strain distribution contour for the vertebrae and the disk for the FSU model at failure.

**Figure 9:**
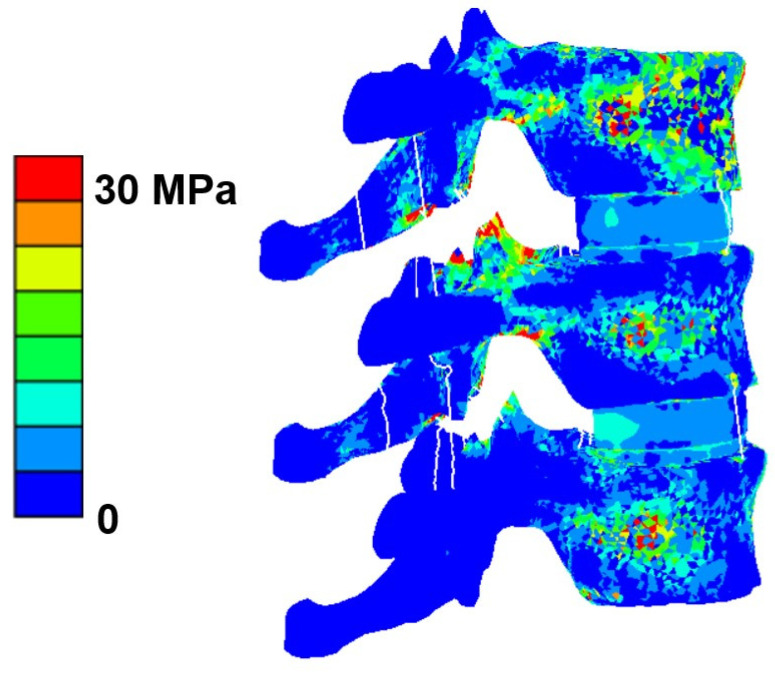
Von Mises stress distribution contour for the vertebrae and the disk for the FSU model at failure.

The mean failure load for the experimental setup was 3513 ± 1029 N, for FSU models with ligaments, it was 2942 ± 943 N, and for FSU models without ligaments, it was 2537 ± 929 N. Figure 4 shows the box plot variation for all three cases. The Spearman correlation coefficient between the FSU with ligament failure loads and experimental loads was 0.79 and for FSU models without ligaments and experimental loads, it was around 0.75. The correlation plots between the FSU model failure loads and experimental loads are shown in Figure 5A,B. BA plots depicting the data spread are shown in Figure 6A,B. Between the experimental failure loads and FSU with ligaments model, the mean value was 576 N, the upper limit (mean + 1.96 SD) was 1268 N, and the lower limit (mean −1.96 SD) was −115 N, respectively. Between the experimental failure loads and FSU without ligaments model, the mean value was 977 N, the upper limit (mean + 1.96 SD) was 1654 N, and the lower limit (mean −1.96 SD) was 300 N, respectively. The vertical displacement contour at the failure is shown in Figure 7. Additionally, the plastic strain distribution for the FSU model at failure is shown in Figure 8. Finally, the von Mises stress distribution is shown in Figure 9.

### 3.2. Comparison of FE-Predicted FSU Failure Load for Models with and without Ligaments

We observed the Spearman correlation value for FSU models with ligaments and without ligaments of 0.99. Figure 5C shows the correlation plot. Figure 6C shows data spread between models with and without ligament through the BA plot. The mean value was 401 N, the upper limit (mean + 1.96 SD) was 610 N, and the lower limit (mean −1.96 SD) was 192 N, respectively. Thus, all these values were positively biased to the FSU with ligament models. We observed a significant difference between the FE failure loads for the FSU models with and without ligaments (*p* value = 0.00054).

## 4. Discussion

In the current study, we compared the failure loads of FSU models from experiments with the FEA-based failure loads derived from the in vitro MDCT data. We observed a reasonably good correlation (ρ = 0.79 and 0.75) between the experimental failure load and FEA-based failure loads for FSU with ligaments. We also observed that the failure loads of the model with ligament showed a narrower data spread than for the model without ligament.

Biomechanically, multiple tissues, including vertebrae, intervertebral disk, ligaments, and other soft tissues, contribute to the structural health of the spine. Analyzing the spine health through the individual vertebrae alone is not sufficient. With the advances in computational capabilities, it is now possible to model and simulate realistic complex biomechanical problems of the spine. Studies have shown that FE-based analysis is a very efficient method for analyzing a single vertebra. Using FE-based methods, researchers have calculated the failure loads and fracture risk more efficiently, compared with the traditional BMD-based methods [17,19,42]. Recently, they have started modeling the functional spine units with at least two vertebrae and one disk. The majority of these studies have concentrated on understanding the biomechanics of the spine [24,25], but fewer studies have concentrated on the computation of bone strength. Calculation of the FSU bone strength is important, as it will give a better idea to clinicians regarding the overall bone health. By using this information, along with other data such as height, weight, etc., the clinicians can determine the critical physical activities for which they can, in turn, offer better care.

There was a good correlation between experimental failure loads and FE-based failure loads for the model with ligaments (ρ = 0.79) and models without ligaments (ρ = 0.75). Groenen et al. simulated the FSU model with patient-specific isotropic material properties identified a strong correlation (R^2^ = 0.68) between the experimental load and FE-based FSU model failure stiffness. They also observed that the correlation between the failure loads is very low (R^2^ = 0.22) [23]. We used the nonlinear patient-specific transversely isotropic material mapping relations for the FSU model in our study. Lee et al. modeled the two vertebrae and one disk FSU model, simulated the forward bending load using FE methodology, and validated the failure load values with experimental results. They observed an excellent correlation up to 0.8–0.87, similar to our study [16]. We also observed that the data spread was higher for the FSU models without ligaments from the BA plot. The mean data spread for with ligaments model was 576 N, and it increased to 977 N for the models without ligaments. From this study, we can conclude that the FSU computational models can be used for the calculation of failure loads. As conducting in vitro biomechanical experimentation is not always possible, computational models provide a good alternative. Additionally, we strongly feel that the methodology used in the current study can be applied to in vivo data, which can further improve osteoporosis diagnosis.

We observed that the presence of ligament had increased the failure load by 404 N on average 16%. Ligaments are connecting tissues that are attached to the bone. These tissues provide flexibility and stability to the spine during motion. There are seven important ligaments in the spine—namely, ALL, PLL, LF, ISL, ITL, SSL, and FCL. All these ligaments connect two vertebrae at multiple locations. Therefore, it is important to understand the effect of ligaments on the failure load value and biomechanical behavior of the spine model. Trajkovski et al. studied the cervical spine ligaments through experimentation and observed that ligaments support within spine mechanical motion. If there is an injury in the ligament, it considerably affects its function [43]. The presence of ligaments can increase the FE problem complexity and, in turn, increase the requirement of computational resources and solving time. In the current study, we sought to understand the effect of ligaments on the FE-predicted failure load for the FSU model. We observed a positive bias of 401 N toward FSU models with ligaments. The observed positive bias shows that the ligaments share the load and support the vertebrae with load transfer. Generally, in many studies, the compression behavior of the ligaments has been neglected. In our study, we observed that the effect of ligaments individually was minimal. Still, when all the ligaments were included with vertebrae, they seemed to influence the FSU failure load values. Therefore, in the current study, we considered only the compression loading for studying the contribution of ligaments on failure load prediction. However, for an accurate understanding of the contributions of ligaments to the spine biomechanics, further studies are warranted, including those with more realistic loading conditions, which is a combination of compression, bending, and twisting experienced by the spine in real-life situations.

The limitations of the current work are as follows: Firstly, the cohort size of 16 patients considered in the current study is relatively small. Secondly, the middle vertebrae were used as the reference to model the upper and bottom vertebrae, and they may have affected the FE failure load. Thirdly, we simplified the disk material model by considering the elastic behavior due to computational resource limitations. In more realistic cases, the annulus of the disk should be modeled as a fiber-reinforced composite [44,45]. We plan to incorporate more complex disk behaviors in future studies. Fourthly, in the current study, we simulated only the axial compression loading. Therefore, when the model is simulated with other loading conditions, the results may vary accordingly. Fifthly, as the tensile load is nominal, we considered similar material behavior for ligaments under both compression and tension; this assumption may have influenced the calculated FE failure load values. In future studies, we aim to incorporate more realistic material behavior for ligaments.

## 5. Conclusions

This study investigated FE-based FSU models with and without ligaments developed from the MDCT data to predict the failure load. We observed good correlations between the FSU models and experimental results. In addition, we also observed that ligaments indeed have an influence on predicting the failure load, even though the contribution is minimal relative to the effort required to model and analyze the functional spinal unit with ligaments. Thus, the inclusion of ligaments in building a simulation model of a functional spine unit for the sole purpose of studying its compression strength is excessive. However, when simulating realistic conditions, which is a combination of compression, bending, and twisting loads experienced by the spine in real-life situations, the contributions of ligaments could be significant due to the nature of load-sharing conditions. Therefore, further research is needed in studying the effect of ligaments in those loading conditions. Finally, the computational modeling may allow the analysis of in vivo data to calculate bone strength, further improving osteoporosis diagnosis and treatment monitoring.

## Figures and Tables

**Table 1:** Image-intensity-based material mapping relations for the vertebrae and material properties for the intervertebral disk used in the current finite element study.

Property	Mapping Relations
**Vertebrae Material Properties**	
Apparent density (ρ_app_ in Kg/m^3^) [29]	ρ_app_ = 47 + 1.122 * HU
Ash density (ρ_ash_ in Kg/m^3^) [30]	ρ_ash_ = 0.6 * ρ_app_
Modulus of elasticity (E in MPa) [29,31]	E_z_ = 4730 * (ρ_app_)^1.56^ E_x_ = E_y_ = 0.333 E_z_ Z- axial direction of the vertebra
Shear modulus (G in MPa) [32]	G_xy_ = 0.121 E_z_ G_xz_ = G_yz_ = 0.157 E_z_
Poisson ratio (V) [32]	Vxy = 0.381 Vxz = Vyz = 0.104
Maximum principal stress limit (σ in MPa) [14]	σ = 137 * ρ_ash_^1.88^, ρ_ash_ < 0.317 σ = 114 * ρ_ash_^1.72^, ρ_ash_ > 0.317
Plastic strain (ε_AB_) [15]	ε_AB_ = -0.00315 + 0.0728 ρ_ash_
Minimum principal stress limit (σ_min_ in MPa) [15]	σ_min_ = 65.1 * ρ_ash_^1.93^
**Intervertebral Disc Properties**	
**Annulus**	
Elastic modulus (E in MPa) [16]	E = 500
Poisson ratio (V) [16]	0.3
Density (ton/mm^3^) [33]	1.2 × 10^−9^
**Nucleus**	
Elastic modulus (E in MPa) [34]	E = 1
Poisson ratio (V) [34]	0.49
Density (ton/mm^3^) [33]	1 × 10^−9^
**Endplate**	
Elastic modulus (E in MPa) [35]	1000
Poisson ratio (V) [35]	0.3
Density (ton/mm^3^) [33]	1 × 10^−9^

**Table 2:** Vertebral ligaments material properties used for functional spine unit model in the current finite element study [33,36].

Name of the Ligament	Density (ton/mm^3^)	Youngs Modulus (MPa)	Poisson’s Ratio	Cicrcular Cross-Sectional Area (mm^2^)	Number of Ligaments
ALL	1 × 10^−9^	55.77	0.4	32.4	3
PLL	1 × 10^−9^	54.43	0.4	05.2	3
LF	1 × 10^−9^	03.25	0.4	84.2	3
ISL	1 × 10^−9^	02.23	0.4	35.1	4
SSL	1 × 10^−9^	12.80	0.4	25.2	2
ITL	1 × 10^−9^	11.50	0.4	12.0	4
FCL	1 × 10^−9^	08.69	0.4	43.8	6

## Data Availability

The raw data supporting the conclusions of this article will be made available by the authors, without undue reservation.

## Abbreviations

3D: three dimensional; FSU: functional spine unit; MDCT: multidetector computed tomography; FE: finite element; IVD: intervertebral disc; DXA: dual-energy X-ray absorptiometry; BMD: bone mineral density; FRAX: Fracture Risk Assessment Tool; QCT: quantitative computed tomography; ALL: anterior longitudinal ligament; PLL: posterior longitudinal ligament; LF: ligamentum flavum; ISL: intraspinous ligament; SSL: supraspinous ligament; ITL: intertransverse ligament; FCL: facet capsular ligament; BA: Bland–Altman.

## References

[1] Frost B.A., Camarero-Espinosa S., Johan Foster E. (2019). Materials for the spine: Anatomy, problems, and solutions. Materials.

[2] Kulkarni V., Akula M., Larouche J. (2018). Current Evaluation and Management of Vertebral Compression Fractures. Curr. Geriatr. Rep..

[3] Adams M.A., Pollintine P., Tobias J.H., Wakley G.K., Dolan P. (2006). Intervertebral disc degeneration can predispose to anterior vertebral fractures in the thoracolumbar spine. J. Bone Miner. Res..

[4] Oner C., Rajasekaran S., Chapman J.R., Fehlings M.G., Vaccaro A.R., Schroeder G.D., Sadiqi S., Harrop J. (2017). Spine Trauma-What Are the Current Controversies?. J. Orthop. Trauma.

[5] Borgström F., Karlsson L., Ortsäter G., Norton N., Halbout P., Cooper C., Lorentzon M., McCloskey E.V., Harvey N.C., Javaid M.K. (2020). Fragility fractures in Europe: Burden, management and opportunities. Arch. Osteoporos..

[6] Hernlund E., Svedbom A., Ivergård M., Compston J., Cooper C., Stenmark J., McCloskey E.V., Jönsson B., Kanis J.A. (2013). Osteoporosis in the European Union: Medical management, epidemiology and economic burden: A report prepared in collaboration with the International Osteoporosis Foundation (IOF) and the European Federation of Pharmaceutical Industry Associations (EFPIA). Arch. Osteoporos..

[7] Faulkner K.G. (2005). The tale of the T-score: Review and perspective. Osteoporos. Int..

[8] Imai K. (2015). Aging and Disease Analysis of Vertebral Bone Strength, Fracture Pattern, and Fracture Location: A Validation Study Using a Computed Tomography-Based Nonlinear Finite Element Analysis. Aging Dis..

[9] Löffler M.T., Sollmann N., Mei K., Valentinitsch A., Noël P.B., Kirschke J.S., Baum T. (2019). X-ray-based quantitative osteoporosis imaging at the spine. Osteoporos. Int..

[10] Schuit S.C.E., Van Der Klift M., Weel A.E.A.M., De Laet C.E.D.H., Burger H., Seeman E., Hofman A., Uitterlinden A.G., Van Leeuwen J.P.T.M., Pols H.A.P. (2004). Fracture incidence and association with bone mineral density in elderly men and women: The Rotterdam Study. Bone.

[11] Kanis J.A., Johnell O., Oden A., Johansson H., McCloskey E. (2008). FRAX^TM^ and the assessment of fracture probability in men and women from the UK. Osteoporos. Int..

[12] Engelke K. (2017). Quantitative Computed Tomography—Current Status and New Developments. J. Clin. Densitom..

[13] Baum T., Muller D., Dobritz M., Wolf P., Rummeny E.J., Link T.M., Bauer J.S. (2012). Converted Lumbar BMD Values Derived from Sagittal Reformations of Contrast-Enhanced MDCT Predict Incidental Osteoporotic Vertebral Fractures. Calcif. Tissue Int.

[14] Keyak J.H., Keller T.S. (1994). Predicting the compressive mechanical behavior of bone. J. Biomech..

[15] Keyak J.H. (2001). Improved prediction of proximal femoral fracture load using nonlinear finite element models. Med. Eng. Phys..

[16] Lee C.H., Landham P.R., Eastell R., Adams M.A., Dolan P., Yang L. (2017). Development and validation of a subject-specific finite element model of the functional spinal unit to predict vertebral strength. Proc. Inst. Mech. Eng. Part H J. Eng. Med..

[17] Imai K., Ohnishi I., Matsumoto T., Yamamoto S., Nakamura K. (2009). Assessment of vertebral fracture risk and therapeutic effects of alendronate in postmenopausal women using a quantitative computed tomography-based nonlinear finite element method. Osteoporos. Int..

[18] Anitha D., Subburaj K., Kopp F.K., Mei K., Foehr P., Burgkart R., Sollmann N., Maegerlein C., Kirschke J.S., Noel P.B. (2019). Effect of Statistically Iterative Image Reconstruction on Vertebral Bone Strength Prediction Using Bone Mineral Density and Finite Element Modeling: A Preliminary Study. J. Comput. Assist. Tomogr..

[19] Allaire B.T., Lu D., Johannesdottir F., Kopperdahl D., Keaveny T.M., Jarraya M., Guermazi A. (2018). Prediction of incident vertebral fracture using CT-based finite element analysis. Osteoporos. Int..

[20] Rayudu N.M., Subburaj K., Mei K., Dieckmeyer M., Kirschke J.S., Noël P.B., Baum T. (2020). Finite Element Analysis-Based Vertebral Bone Strength Prediction Using MDCT Data: How Low Can We Go?. Front. Endocrinol..

[21] Yeung L.Y., Rayudu N.M., Löffler M., Sekuboyina A., Burian E., Sollmann N., Dieckmeyer M., Greve T., Kirschke J.S., Subburaj K. (2021). Prediction of Incidental Osteoporotic Fractures at Vertebral-Specific Level Using 3D Non-Linear Finite Element Parameters Derived from Routine Abdominal MDCT. Diagnostics.

[22] Anitha D.P., Baum T., Kirschke J.S., Subburaj K. (2019). Effect of the intervertebral disc on vertebral bone strength prediction: A Finite-Element study. Spine J..

[23] Groenen K.H.J., Bitter T., van Veluwen T.C.G., van der Linden Y.M., Verdonschot N., Tanck E., Janssen D. (2018). Case-specific non-linear finite element models to predict failure behavior in two functional spinal units. J. Orthop. Res..

[24] Xiao Z., Wang L., Gong H., Zhu D., Zhang X. (2011). A non-linear finite element model of human L4-L5 lumbar spinal segment with three-dimensional solid element ligaments. Theor. Appl. Mech. Lett..

[25] Jahng T.A., Kim Y.E., Moon K.Y. (2013). Comparison of the biomechanical effect of pedicle-based dynamic stabilization: A study using finite element analysis. Spine J..

[26] Baum T., Gräbeldinger M., Räth C., Grande Garcia E., Burgkart R., Patsch J.M., Rummeny E.J., Link T.M., Bauer J.S. (2014). Trabecular bone structure analysis of the spine using clinical MDCT: Can it predict vertebral bone strength?. J. Bone Miner. Metab..

[27] Anitha D., Thomas B., Jan K.S., Subburaj K. (2017). Risk of vertebral compression fractures in multiple myeloma patients: A finite-element study. Medicine.

[28] O’Connell G.D., Vresilovic E.J., Elliott D.M. (2007). Comparison of animals used in disc research to human lumbar disc geometry. Spine.

[29] Rho J.Y., Hobatho M.C., Ashman R.B. (1995). Relations of mechanical properties to density and CT numbers in human bone. Med. Eng. Phys..

[30] Goulet R.W., Goldstein S.A., Ciarelli M.J., Kuhn J.L., Brown M.B., Feldkamp L.A. (1994). The relationship between the structural and orthogonal compressive properties of trabecular bone. J. Biomech..

[31] Morgan E.F., Bayraktar H.H., Keaveny T.M. (2003). Trabecular bone modulus-density relationships depend on anatomic site. J. Biomech..

[32] Crawford R.P., Cann C.E., Keaveny T.M. (2003). Finite element models predict in vitro vertebral body compressive strength better than quantitative computed tomography. Bone.

[33] Sivasankari S., Balasubramanian V. (2021). Influence of occupant collision state parameters on the lumbar spinal injury during frontal crash. J. Adv. Res..

[34] Ayturk U.M., Puttlitz C.M. (2011). Parametric convergence sensitivity and validation of a finite element model of the human lumbar spine. Comput. Methods Biomech. Biomed. Engin..

[35] Lv Q.B., Gao X., Pan X.X., Jin H.M., Lou X.T., Li S.M., Yan Y.Z., Wu C.C., Lin Y., Ni W.F. (2018). Biomechanical properties of novel transpedicular transdiscal screw fixation with interbody arthrodesis technique in lumbar spine: A finite element study. J. Orthop. Transl..

[36] Khoz Z., Nikkhoo M., Cheng C. (2018). Parametric Patient-Specific Finite Element Modeling of Lumbar Spine Based on Anatomical Parameters. Iran. J. Orthop. Surg..

[37] Li J., Shang J., Zhou Y., Li C., Liu H. (2015). Finite element analysis of a new pedicle screw-plate system for minimally invasive transforaminal lumbar interbody fusion. PLoS ONE.

[38] Anitha D., Mei K., Dieckmeyer M., Kopp F.K., Sollmann N., Zimmer C., Kirschke J.S., Noel P.B., Baum T., Subburaj K. (2019). MDCT-based Finite Element Analysis of Vertebral Fracture Risk: What Dose is Needed?. Clin. Neuroradiol..

[39] Anitha D., Subburaj K., Mei K., Kopp F.K., Foehr P., Noel P.B., Kirschke J.S., Baum T. (2016). Effects of dose reduction on bone strength prediction using finite element analysis. Sci. Rep..

[40] Dall’Ara E., Pahr D., Varga P., Kainberger F., Zysset P. (2012). QCT-based finite element models predict human vertebral strength in vitro significantly better than simulated DEXA. Osteoporos. Int..

[41] Bland J.M., Altman D.G. (1999). Measuring agreement in method comparison studies. Stat. Methods Med. Res..

[42] Kopperdahl D.L., Aspelund T., Hoffmann P.F., Sigurdsson S., Siggeirsdottir K., Harris T.B., Gudnason V., Keaveny T.M. (2014). Assessment of incident spine and hip fractures in women and men using finite element analysis of CT scans. J. Bone Miner. Res..

[43] Trajkovski A., Hribernik M., Kunc R., Kranjec M., Krašna S. (2020). Analysis of the mechanical response of damaged human cervical spine ligaments. Clin. Biomech..

[44] Park W.M., Kim C.H., Kim Y.H., Chung C.K., Jahng T.A. (2015). The change of sagittal alignment of the lumbar spine after dynesys stabilization and proposal of a refinement. J. Korean Neurosurg. Soc..

[45] Zhong Z.C., Wei S.H., Wang J.P., Feng C.K., Chen C.S., Yu C.H. (2006). Finite element analysis of the lumbar spine with a new cage using a topology optimization method. Med. Eng. Phys..

